# P70 Rising antibiotic resistance in respiratory samples of hospitalized patients—an alarm to rise up and prevent the upcoming threat

**DOI:** 10.1093/jacamr/dlaf230.077

**Published:** 2025-12-04

**Authors:** Madhavi Kirti, Parul Singh, M Nizam Ahmed, Vanlal Tluanpuii, Bharat Chandra Das, Purva Mathur

**Affiliations:** Department of Microbiology, JPNATC, AIIMS, New Delhi, India; Department of Laboratory Medicine, JPNATC, AIIMS, New Delhi, India; Department of Microbiology, JPNATC, AIIMS, New Delhi, India; Department of Microbiology, JPNATC, AIIMS, New Delhi, India; Department of Microbiology, JPNATC, AIIMS, New Delhi, India; Department of Laboratory Medicine, JPNATC, AIIMS, New Delhi, India

## Abstract

**Background:**

Respiratory tract infections cause significant morbidity and mortality globally and are the most common infectious diseases in hospitalized patients. There is a wide variety of respiratory diseases included in the hospital-acquired lower respiratory tract infections (HA-LRTIs) with different causative pathogens, and current treatment is essentially empirical, often resulting in an inaccurate use of antibiotics. The use of antibiotic treatment without certain knowledge of the causative pathogen can lead to treatment failure and even the development of resistances. As a result, along with the threatening growth of antibiotic resistance, a decrease in the patient’s health status and a rise in treatment costs may occur.

**Methods and materials:**

We have carried out this study for the period of 6 months, July 2024–December 2024 in JPNATC, AIIMS, New Delhi. The respiratory samples for the study were Broncho-alveolar lavage (BAL), tracheal aspirate and sputum. The samples were processed for culture and its antibiotic sensitivity pattern in conventional as well as in automated manner to look for the bacterial infections and their antimicrobial susceptibility pattern. The samples were collected from the patients who have been admitted for more than one week.

**Results:**

Total respiratory samples collected were 420. The leading bacteria was found to be *Acinetobacter baumannii* (48.5%) either with XDR pattern or resistance to carbapenems. The other leading organisms were *Klebsiella pneumoniae, Pseudomonas aeruginosa, Escherichia coli, Stenotrophomonas maltophilia, Providencia stuartii, Staphylococcus aureus, Burkholderia cepacia, Serratia marcescens*, *Streptococcus pneumoniae, Proteus mirabilis and Candida albicans.* All the isolates showed highly resistant pattern for cephalosporins, carbapenems and/or all groups of drugs, leaving minimum options for treatment like colistin and vancomycin.

**Conclusions:**

This survey aims to describe hospital acquired lower respiratory tract infections and their antibiotic susceptibility pattern giving an alarm for rising antibiotic resistance pattern in patients admitted to the hospital.

